# Imperatorin induces autophagy and G0/G1 phase arrest via PTEN-PI3K-AKT-mTOR/p21 signaling pathway in human osteosarcoma cells in vitro and in vivo

**DOI:** 10.1186/s12935-021-02397-7

**Published:** 2021-12-19

**Authors:** Minchao Lv, Qingxin Xu, Bei Zhang, Zhiqiang Yang, Jun Xie, Jinku Guo, Feixiong He, Wei Wang

**Affiliations:** 1 Department of Orthopedics, Quzhou People’s Hospital, The Quzhou Affiliated Hospital of Wenzhou Medical University, No.100, Minjiang Avenue, Quzhou, Zhejiang China; 2 Department of Clinical Medicine, Second Clinical Medical College, Wenzhou Medical University, Chashan Educational District, Wenzhou, Zhejiang China; 3 First Clinical Medicine College, Zhejiang Chinese Medical University, No. 548, Bingwen Road, Hangzhou, Zhejiang China; 4 Department of Orthopedics, Zhongnan Hospital of Wuhan University, No. 169, Donghu Road, Wuhan, Hubei China

**Keywords:** Imperatorin, Osteosarcoma, PTEN-PI3K-AKT-mTOR/p21 pathway, Cell cycle, Autophagy

## Abstract

**Background:**

Osteosarcoma is the third most common cancer in adolescence and the first common primary malignant tumor of bone. The long-term prognosis of osteosarcoma still remains unsatisfactory in the past decades. Therefore, development of novel therapeutic agents which are effective to osteosarcoma and are safe to normal tissue simultaneously is quite essential and urgent.

**Methods:**

Firstly, MTT assay, cell colony formation assay, cell migration and invasion assays were conducted to evaluate the inhibitory effects of imperatorin towards human osteosarcoma cells. RNA-sequence assay and bioinformatic analysis were then performed to filtrate and assume the potential imperatorin-induced cell death route and signaling pathway. Moreover, quantitative real-time PCR assay, western blot assay and rescue experiments were conducted to confirm the assumptions of bioinformatic analysis. Finally, a subcutaneous tumor-transplanted nude mouse model was established and applied to evaluate the internal effect of imperatorin on osteosarcoma by HE and immunohistochemistry staining.

**Results:**

Imperatorin triggered time-dependent and dose-dependent inhibition of tumor growth mainly by inducing autophagy promotion and G0/G1 phase arrest in vitro and in vivo. Besides, imperatorin treatment elevated the expression level of PTEN and p21, down-regulated the phosphorylation of AKT and mTOR. In contrast, the inhibition of PTEN using Bpv (HOpic), a potential and selective inhibitor of PTEN, concurrently rescued imperatorin-induced autophagy promotion, cell cycle arrest and inactivation of PTEN-PI3K-AKT-mTOR/p21 pathway.

**Conclusions:**

This work firstly revealed that imperatorin induced autophagy and cell cycle arrest through PTEN-PI3K-AKT-mTOR/p21 signaling pathway by targeting and up-regulating PTEN in human osteosarcoma cells. Hence, imperatorin is a desirable candidate for clinical treatments of osteosarcoma.

**Supplementary Information:**

The online version contains supplementary material available at 10.1186/s12935-021-02397-7.

## Background

Osteosarcoma (OS), an orphan disease with an overall incidence of 0.2–3/10^5^ while 0.8–11/10^5^ in the age group of 15–19 years, has been reported to be the third most common cancer in adolescence and account for around 60% of all bone cancer [1, 2]. It always brings the patients great sufferings, lifelong disability and severe mental trauma. At the very beginning, radical operation without further treatments could only cure about 20% of the patients due to the early distant metastasis, which could be found in 20% patients at the time of diagnosis [3, 4]. Nowadays, the combination of radical operation and advanced radio/chemotherapy afterwards, if feasible, have promoted the 5-year survival rate to 70% for localized tumors [4, 5]. However, due to the high recurrence rate, the prognosis still remains unsatisfactory for those with unresectable or metastatic cancers [4]. Therefore, development of novel therapeutic agents which are effective to osteosarcoma and are safe to normal tissue simultaneously is quite essential and urgent.

Imperatorin (IMP), a bioactive coumarin compound mainly extracted from *Angelica dahurica* and *Angelica sinensis*, which have a widespread application in traditional Chinese and Japanese medicine for the treatment of headache, rhinitis and cardiovascular diseases etc. [6, 7]. It has been reported that IMP possessed wide pharmacological activities, such as neuroprotective, anti-hypertension, antibacterial, anti-inflammatory and anti-cancer properties [8–10]. Furthermore, a large number of in vitro and in vivo researches demonstrated that IMP possessed the significant suppressive effects towards hepatoma [11, 12], colon cancer [13, 14], cervical cancer [15], gastric carcinoma [16], lung cancer [17], melanoma [18] and breast cancer [19] through a time- and dose-dependent manner. The researchers also found that IMP combined with other relevant anti-cancer drugs worked better in vitro against many kinds of carcinomas [10, 12, 20, 21]. Besides, researchers also explored the possible toxicity of IMP, which suggested that the moderate concentration of IMP treatment did not cause much cytotoxicity to mouse fibroblast cells [16]. Owing to its widespread anti-cancer activity, satisfactory biocompatibility as well as the potential to work with other anti-cancer drugs, IMP is possible to be an attractive novel agent in the clinical treatment of osteosarcoma, which makes it very necessary to investigate the antitumor effects and molecular mechanisms of IMP on human osteosarcoma cells.

Autophagy, which is considered to be an important factor when evaluating anti-cancer drugs, delivers cytoplasmic contents to lysosomes and degraded the contents through double-membrane vesicles [22]. Microtubule-associated protein 1A/1B-light chain 3 (LC3), a molecular closely related to autophagy, is widely distributed in mammal cells [23]. During autophagy progression, after being conjugated with phosphatidylethanolamine, the cytosolic form of LC3 (LC3-I) transforms to LC3-phosphatidylethanolamine (LC3-II) at autophagosomal membranes [24]. Therefore, the activity level of autophagy can be determined by immunodetecting the ratio of LC3-II–LC3-I.

Cell cycle is a conserved progression precisely controlled by a regulatory network [25]. The accurate transition from G0/G1 phase to S phase is crucial for the control of cell proliferation, while the derepression of G1-S transcription allows cells to unrestrainedly process into S phase and consequently triggers oncogenesis [25, 26]. It has been reported that human cells progressed into a new cell cycle during G1 phase via activating Cyclin-CDK-dependent transcription (especially CDK4-Cyclin D1 and CDK6-Cyclin D1), which indicated that the G1 phase arrest can be monitored by the immunodetecting of the expression level of CDK6 and Cyclin D1 [25].

As for the underlying molecular mechanisms, it has been reported that mammalian target of rapamycin (mTOR) not only is a major negative regulator of cellular autophagy, but also participates in cell proliferation regulation [27, 28]. Furthermore, the phosphorylation of mTOR is regulated by the phosphoinositide-3 kinase (PI3K)-mediated activation of protein kinase B (AKT) [29]. With regard to the regulation of cell cycle, p21 is a kind of CDK inhibitors, which was uniquely involved in maintaining the G1 cell cycle arrest [30]. Moreover, p21 was also discovered as a highly regulated gene with many pathways such as the PI3K-AKT signaling pathway.

In this study, we attempted to systematically evaluate the suppressive effects of IMP on human OS cells in vitro and in vivo. Furthermore, we explored and confirmed the molecular mechanisms of its inhibitory effects on osteosarcoma growth mainly by RNA-seq, qRT-PCR and Western blot assays, which is, induction of autophagy and G0/G1 phase arrest by inactivating the PTEN-PI3K-AKT-mTOR/p21 signaling pathway via targeting and up-regulating the expression of PTEN.

## Methods

### Antibodies and reagents

Imperatorin (98%, HPLC) was purchased from Shanghai Aladdin Biochemical Technology Co., Ltd. (China). The PTEN inhibitor BpV (HOpic) was obtained from MedChemExpress LLC (USA). Roswell Park Memorial Institute 1640 (RMPI-1640) medium and Foetal Bovine Serum (FBS) were obtained from Gibco Life Technologies (USA). Phosphate-buffered saline (PBS) was purchased from Meilunbio Co., Ltd. (China). 3-(4,5-dimethylthiazol-2-yl)-2,5-Diphenyltetrazolium bromide (MTT), Crystal Violet Staining Solution, DAPI staining solution, Propidium Iodide together with Cell lysis buffer for Western and IP were all purchased from Beyotime Biotechnology (China). TRIzol reagent was obtained from Invitrogen (USA). TB Green^®^ Premix Ex Taq™ (Tli RNaseH Plus) and PrimeScript™ RT Master Mix (Perfect Real Time) were purchased from Takara Biomedical Technology (Japan). Acridine orange (AO) staining solution was obtained from Sigma-Aldrich Co., LLC (USA). 5 ×  protein loading buffer was obtained from ASPEN Biotechnology CO., Ltd. (China). Primary antibodies, including GAPDH, CDK6, Cyclin D1, ULK1, LC3B, ATG5, PI3K (p85 subunit), p-PI3K (p-p85 subunit), AKT, p-AKT, mTOR, p-mTOR, together with secondary antibodies were all purchased from Cell Signaling Technology (USA). Primary antibody against p21 was obtained from Abcam (USA). ECL chemiluminescent substrate kit (super-sensitive) were purchased from Biosharp Life Science Co., Ltd. (China).

### Cell culture

Human osteosarcoma cells 143B (ATCC CRL-8303) and U2OS (ATCC HTB-96) were purchased from National Collection of Authenticated Cell Cultures (Shanghai, China) at July, 2020. The cell lines have been authenticated by STR (Short Tandem Repeat) authentication and tested for mycoplasma contamination by National Collection of Authenticated Cell Cultures (Shanghai, China) before the purchase. The 143B cells were cultured in RMPI-1640 medium supplemented with 10% (v/v) FBS and 1% (v/v) penicillin/streptomycin. The U2OS cells were cultured in Mccoy’s 5A medium supplemented with 10% (v/v) FBS and 1% (v/v) penicillin/streptomycin. The cells were maintained in an incubator at 37 ℃ with 5% CO_2_. The medium was replaced every 2 days during the culture.

### Cell proliferation assay

MTT assay was conducted to evaluate the effects of IMP on the proliferation of OS cells. In brief, 143B or U2OS cells were seeded in 96-well plates at a density of 2 × 10^3^ or 4 × 10^3^ cells per well with 200 μL medium, respectively, followed by being incubated at 37 ℃, 5% CO_2_ for 24 h. Then, the cells were exposed to fresh medium with various concentration of IMP for 24 and 48 h. At the end of the treatment, 20 μL of sterile MTT solution (0.5%) was added to each well and the plate was incubated for another 4 h at 37 ℃. After that, the medium in each well was replaced by 150 μL DMSO and an PE Enspire multimode microplate reader (PerkinElmer, USA) was used to measure the absorbance of each well at 570 nm.

### Cell colony formation assay

The OS cells were seeded in six-well plates with a density of 5 × 10^2^ cells per well and were incubated at 37 ℃, 5% CO_2_ for about 7 days until the colonies were clearly visible. In the IMP treatment group, cells were treated with fresh medium containing various concentration of IMP. The culture medium was replaced every 2–3 days during the incubation. Then, the colonies were fixed with 4% paraformaldehyde and subsequently stained by crystal violet solution for 15 min. In the end, the number of colonies were photographed and calculated.

### Cell invasion assay

The invasion ability of OS cells was determined using 24-well Boyden chambers with 8 μm pore size pre-coated with Matrigel as described previously [31]. Briefly, the OS cells were pre-treated with various concentration of IMP for 24 h. After that, the cells were digested, resuspended in serum-free medium and subsequently seeded in the upper chamber with a density of 2 × 10^4^ cells per chamber. 800 μL of fresh medium with 10% FBS was added to the lower chamber. After the incubation at 37 ℃, 5% CO_2_ for 24 h, the cells invaded to the lower surface of the chamber were fixed by 4% paraformaldehyde, stained by crystal violet and photographed under an inverted microscope. Invaded cells of 5 fields (100 ×) were observed and counted for each chamber. The cell invasion assay was performed independently in triplicate.

### Cell migration assay

The migration ability of OS cells was measured by wound-healing migration assay. Generally, the cells were seeded in six-well plates at a density of 3 × 10^5^ cells per well and cultured at 37 ℃, 5% CO_2_ for 24 h. After the cells growing to a confluent monolayer, the “wound” was carefully created in the well with a sterile pipette tip. The cells were subsequently washed with PBS twice and incubated in fresh serum-free medium with various concentration of IMP for 24 h. The cells were photographed with an inverted microscope at 0 and 24 h after the IMP treatment.

### Total RNA extraction

Total RNA was isolated and purified using TRIzol reagent following the manufacturer's procedure. The total RNA concentration and purity of each sample was quantified using NanoDrop ND-1000 (NanoDrop, USA).

### RNA-seq assay and bioinformatic analysis

The RNA-seq assay was performed by LC Bio Technology CO., Ltd. (Hangzhou, China). Briefly, the cDNA library (300 ± 50 bp) was prepared for sequencing using the standard Illumina protocols. Then, the 2 × 150 bp paired-end sequencing (PE150) was performed on an Illumina Novaseq™ 6000 (Illumina, USA) following the vendor’s recommended protocol. The differentially expressed gene analysis was selected with fold change  > 2 or  < 0.5 and p value  < 0.05 by R package edgeR. Gene Ontology (GO) enrichment analysis was performed by R package GOseq. The Kyoto Encyclopedia of Genes and Genomes (KEGG) enrichment analysis was also conducted to determine the pathway enrichment. The results of both GO and KEGG enrichment analysis were considered statistically significant when the adjusted p value was less than 0.05. Besides, the visualized KEGG pathway heatmaps with relative gene expression level were established by R package Pathview.

### Quantitive real time PCR

cDNA was synthesized from 500 μg of total RNA sample by reverse transcription with PrimeScript™ RT Master Mix. qRT-PCR reaction was conducted on a Roche LightCycler 480 Instrument II (Roche, USA) with TB Green^®^ Premix Ex Taq™. The primers sequences used in qRT-PCR reaction were shown in Additional file 1: Table S1. Data were analyzed using the comparative Ct method (2^−ΔΔCt^).

### Cell cycle analysis by flow cytometry

The OS cells were seeded in six-well plate with a density of about 1 × 10^5^ cells per well and then treated with various concentration of IMP for 24 h. After the treatment, the cells were digested, washed by cold PBS for 2 times, stained with propidium iodide (PI) and finally analyzed by a Cytoflex S (Beckman Coulter, USA). The flow cytometry data was further analyzed by Modfit LT software to evaluate the distribution of the cells throughout G0/G1, S, G2/M phases of the cell cycle.

### Cell autophagy assay (AO staining)

In order to evaluate the autophagy level of OS cells, acidic vesicular organelles (AVOs) formation, a morphological characteristic of cell autophagy, was detected by acridine orange (AO) staining as preciously described [32]. In brief, OS cells were stained with 1 μg/mL AO solution for 20 min, washed with PBS for three times, followed by being observed under a laser scanning microscope with the wavelength channel of 546 nm and 575/640 nm for excitation and emission, respectively.

### Cell autophagy assay (LC3B immunofluorescence)

Immunofluorescence staining of LC3B (a reliable marker of autophagy activation) was also conducted to detect the autophagy level of OS cells. Briefly, IMP treated cells were fixed with 4% paraformaldehyde followed by being washed with PBS for 3 times. Then, the samples were successively incubated with 0.2% Triton X-100 (5 min), blocking buffer (1 h), primary antibody (2 h at room temperature), fluorochrome-labeled secondary antibody (1 h), and DAPI staining solution (15 min). Finally, the samples were observed under a laser scanning microscope.

### Western blotting analysis

OS cells were seeded at a density of 1.5 × 10^5^ cells per well in six-well plates and treated with various concentration of IMP for 24 h. After the treatment, the cells were digested, washed with cold PBS and lysed by ice-cold Cell lysis buffer for Western and IP containing 1% protease and phosphatase inhibitor(100 ×) for 30 min on ice. Then, the cell lysates were centrifuged at 12,800 rpm for 15 min at 4 ℃ and the supernatant was collected carefully. The protein solution was then added with protein loading buffer and denatured at 95 ℃ for 5 min. Protein concentration was quantified using the BSA Kit according to the manufacturer’s instruction. Equal amounts of total protein samples were separated by SDS-PAGE (6–15%) gel electrophoresis at 80 V for 45 min and then 120 V for 45 min, and subsequently transferred to 0.45 μm PVDF membrane at 200 mA (250 mA for mTOR and p-mTOR) for 90 min. After being blocked by 5% skim milk in TBST buffer for 1.5 h at room temperature, the PVDF membranes were incubated with relative primary antibody overnight at 4 ℃. After the incubation, the membranes were washed three times with TBST buffer and then incubated with the HRP-conjugated secondary antibody for 1 h at room temperature. At the end, the protein expression level was detected with ECL chemiluminescent substrate kit by a Quickchemi 5200 Chemiluminescence Imaging System (Monad, China) following the vendor’s instructions.

### Animal experiments

All experiments on live animals in this work were evaluated and approved by Institutional Animal Care and Use Committee of Wuhan University (AUP No. WP2020-08106). To evaluate the anti-tumor effect of IMP in vivo, 10 female SPF BALB/c-nu mice were purchased at age of 4 weeks in a standard animal laboratory with free access to food and water. 143B cells were digested, washed by cold PBS three times and resuspended with cold PBS at a density of 1 × 10^7^ cells/mL. Hundred μL of the cell suspension was injected subcutaneously into the axilla of each mouse. When the tumors were visible, the mice were randomly divided into two groups: the control group and the IMP group (five mice in each group). The mice in control group were intraperitoneally injected of 100 μL saline with 5% DMSO every other day, while that in IMP group were intraperitoneally injected of 100 μL IMP solution (5 mg/kg, diluted with 5% DMSO saline solution) at the same time. Tumor volume and mice bodyweight were measured and recorded before each injection. After 5 times of drug injection, the mice were sacrificed and its tumors, heart, liver, spleen, lung and kidney were isolated and fixed for histopathology and immunohistochemistry (IHC) experiments. To evaluate the biocompatibility of IMP in vivo, the heart, liver, spleen, lung and kidney sections were stained with H&E and scanned. To confirm the anti-tumor effects and its molecular mechanisms, H&E staining and the IHC assays of PTEN, p-AKT, CDK6 and LC3 were performed and scanned on tumor tissue sections. Furthermore, the technical details of the euthanasia of animals were provided as below: Firstly, all the animals were properly anesthetized by inhaling isoflurane (gas flow rate: 400 mL·min^−1^, induced concentration: 3%) through an inhaled anesthesia machine (Matrx, USA). The treated time was determined by animal behavior. The animals were considered being anesthetized adequately when it turns over and does not return to the prone posture, generally it’s 2–4 min. Then, the anesthetized animals were sacrificed by breaking spine method.

### Statistical analysis

In this work, statistical analysis was performed by unpaired t test, one-way or two-way analyses of variance (ANOVA) according to the experimental design. Also, posthoc analysis using the Tukey method was applied to determine multiple comparisons in ANOVA analysis (GraphPad Prism 7.02). Statistical significance was attained with greater than 95% confidence level (p  < 0.05).

## Results

### IMP inhibited the proliferation, migration and invasion of osteosarcoma in vitro

**Fig. 1:**
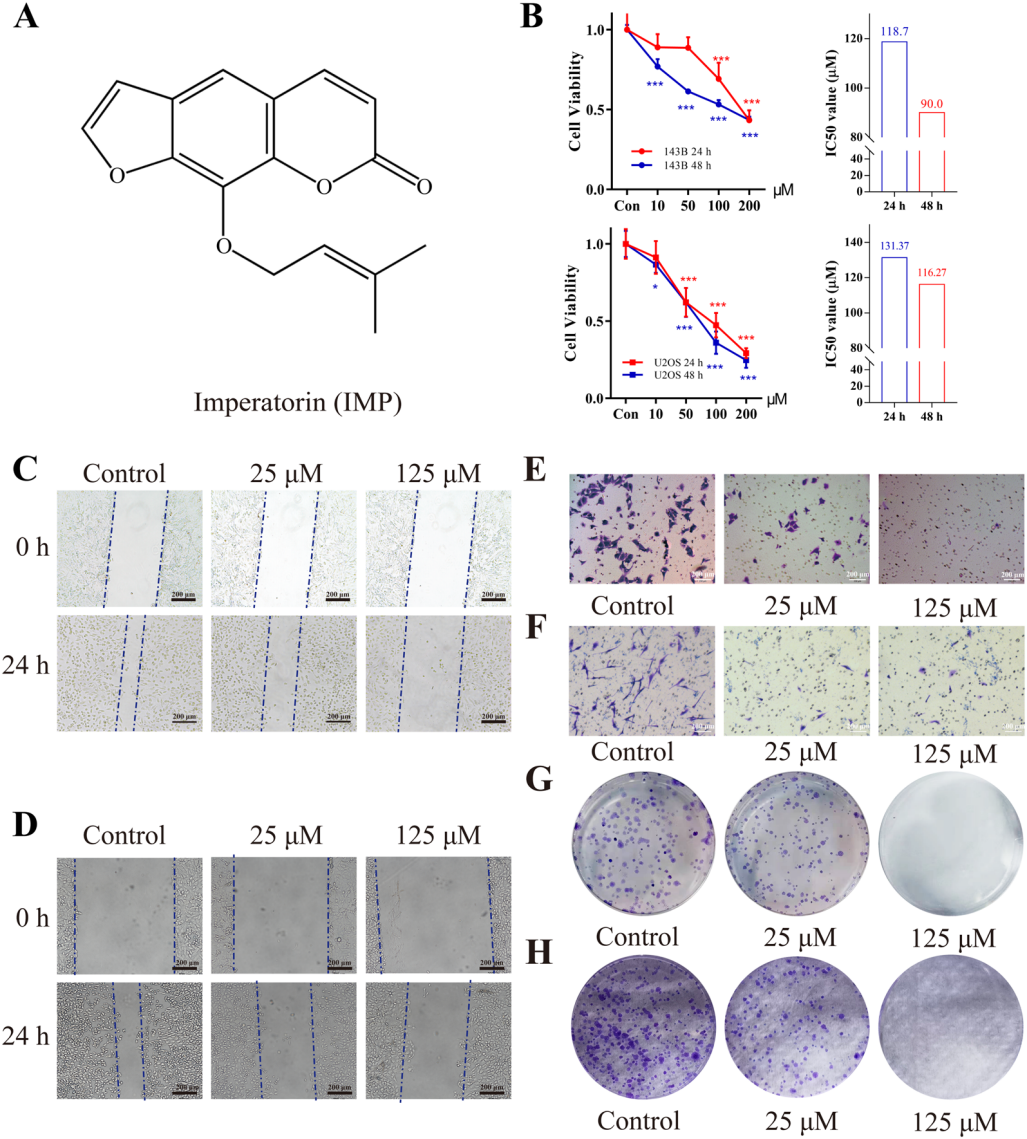
IMP depressed the proliferation, invasion and migration of human osteosarcoma cells. **A** Chemical structure of IMP. **B** IMP inhibited the proliferation of 143B and U2OS cells in a dose-dependent and time-dependent manner. IMP repressed the migration of 143B (**C**) and U2OS (**D**) cells in a dose-dependent manner. IMP inhibited the invasion of 143B (**E**) and U2OS (**F**) cells in a dose-dependent manner. IMP inhibited colony formation of 143B (**G**) and U2OS (**H**) cells in a dose-dependent manner. Scale bars equaled 200 μm. *p  < 0.05; **p  < 0.01; ***p  < 0.001, one-way ANOVA; data were mean  ±  SD

To evaluate the effect of IMP (Fig. 1A showed its molecular structure) on the proliferation of OS cells, MTT assays, IC50 value calculation and colony formation assays were conducted for 143B and U2OS cells treated with various concentration of IMP. The MTT results (Fig. 1B) suggested that IMP significantly inhibited the proliferation of OS cells with a dose-dependent manner in both 24 h and 48 h experiments (p  < 0.05). The IC50 values (Fig. 1B) of IMP on 143B cells were 118.7 μM and 90.0 μM for 24 and 48 h treatment, respectively, while that on U2OS cells were 131.4 μM and 116.3 μM for 24 and 48 h treatment, respectively. Moreover, the results of colony formation assays (Fig. 1G, H) demonstrated that IMP treatment significantly reduced the colony formation counts of both 143B and U2OS cells in a dose-dependent manner. These results indicated that IMP inhibited the proliferation of OS cells in a dose- and time-dependent manner.

To assess the effect of IMP on the migration and invasion ability of OS cells, “wound-healing” and transwell experiments were performed on OS cells exposed to various concentration of IMP for 24 h, respectively. The results of wound-healing assay (Fig. 1C, D) and transwell experiment (Fig. 1E, F) demonstrated that IMP significantly suppressed the migration and invasion of OS cells in a dose-dependent manner.

### RNA-sequence assay and bioinformatic analysis

**Fig. 2:**
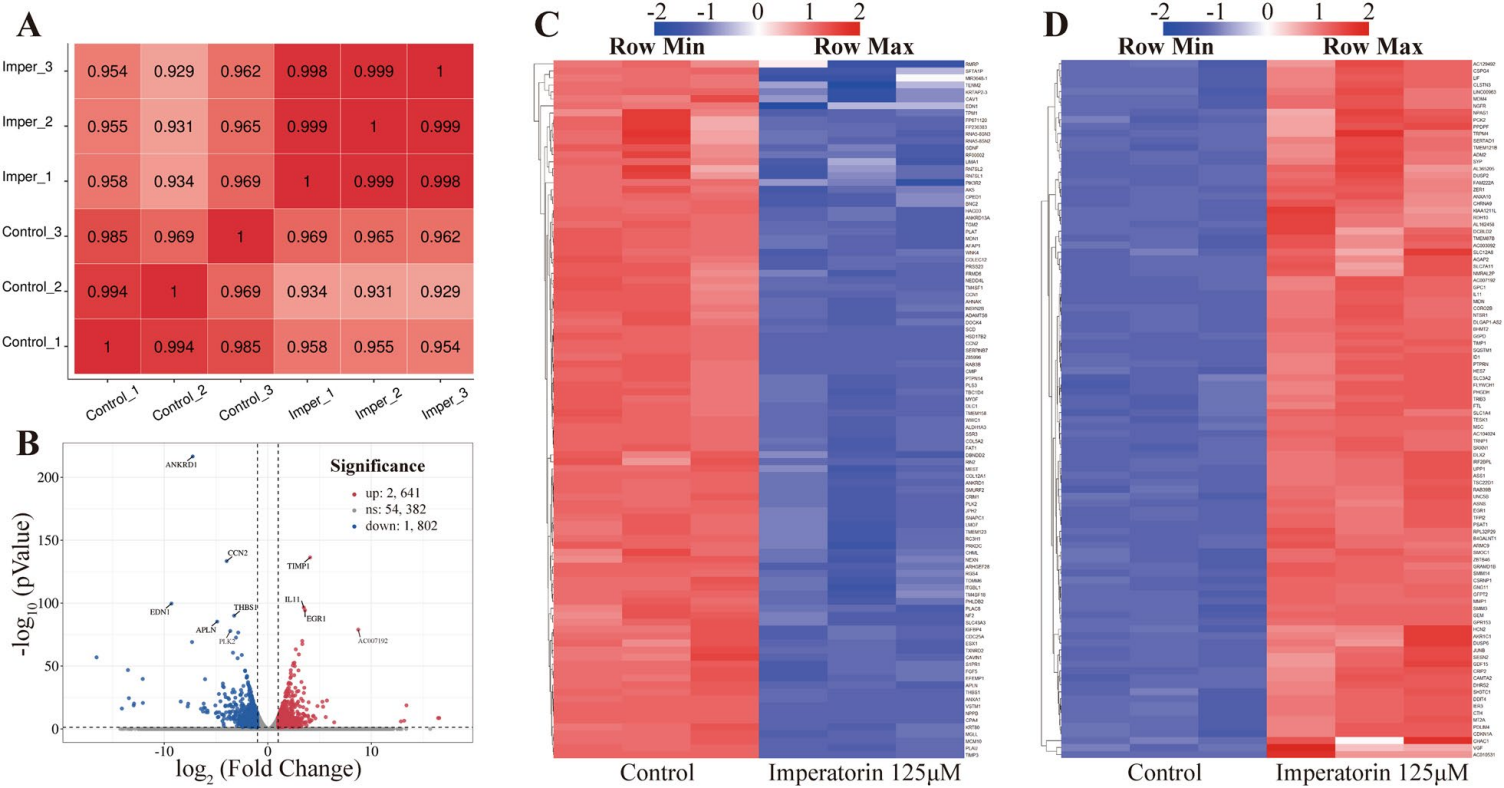
The overview of RNA-seq data. **A** Pearson correlation map between each sample of RNA-seq. **B** Volcano map of differential expressed genes after IMP treatment. **C** Heatmap of top 100 down-regulating expressed genes after IMP treatment. **D** Heatmap of top 100 up-regulating expressed genes after IMP treatment

To further investigate the molecular mechanisms behind the IMP’s inhibitory effects on the OS cells, RNA-seq assay was performed on 143B cells exposed to 125 μM IMP and blank control, respectively. In brief, as shown in Fig. 2, IMP treatment significantly up-regulated the expression level of 2641 genes and down-regulated that of 1802 genes in 143B cells, compared with the control group.

**Fig. 3:**
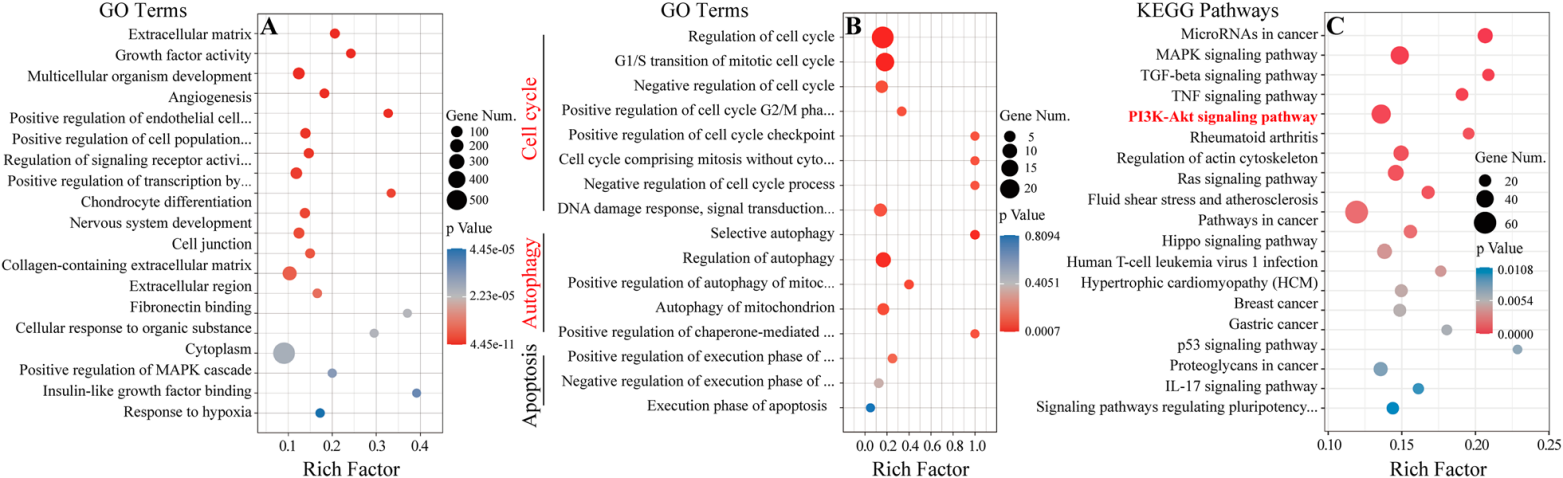
Bioinformatic analysis of RNA-seq data. **A **The scatterplot image of top 20 altered GO terms after IMP treatment in GO enrichment results. **B** The scatterplot image of GO terms relevant with cell cycle, autophagy and apoptosis in GO enrichment results. **C** The scatterplot image of top 20 altered KEGG pathways after IMP treatment in KEGG enrichment results

Then, GO and KEGG enrichment analysis were performed to identify the signaling pathways possibly influenced by IMP treatment. Figure 3A showed the top 20 altered GO terms in IMP-treated 143B cells. As we know, the mechanisms of proliferation inhibition to OS could be roughly divided into three main types: apoptosis, autophagy and cell cycle arrest. Figure 3B illustrated the GO terms relevant with apoptosis, autophagy and cell cycle, respectively, which demonstrated that nearly all GO terms relevant to autophagy and cell cycle were significantly altered after the IMP treatment, while there is almost no change in GO terms relevant with apoptosis. These results suggested that IMP treatment possibly inhibited the proliferation of OS cells mainly via the promotion of autophagy and cell cycle arrest. Figure 3C showed the top 20 altered signaling pathways enriched by KEGG analysis in IMP-treated 143B cells. PI3K-AKT signaling pathway was one of the top 20 enriched pathways and was strongly relevant to the regulation of autophagy and cell cycle of OS cells. Therefore, these results suggested that IMP possibly inhibited the proliferation of OS cells via the induction of autophagy and cell cycle arrest by regulating PI3K-AKT signaling pathway.

### IMP induced G0/G1 phase arrest of cell cycle in vitro

**Fig. 4:**
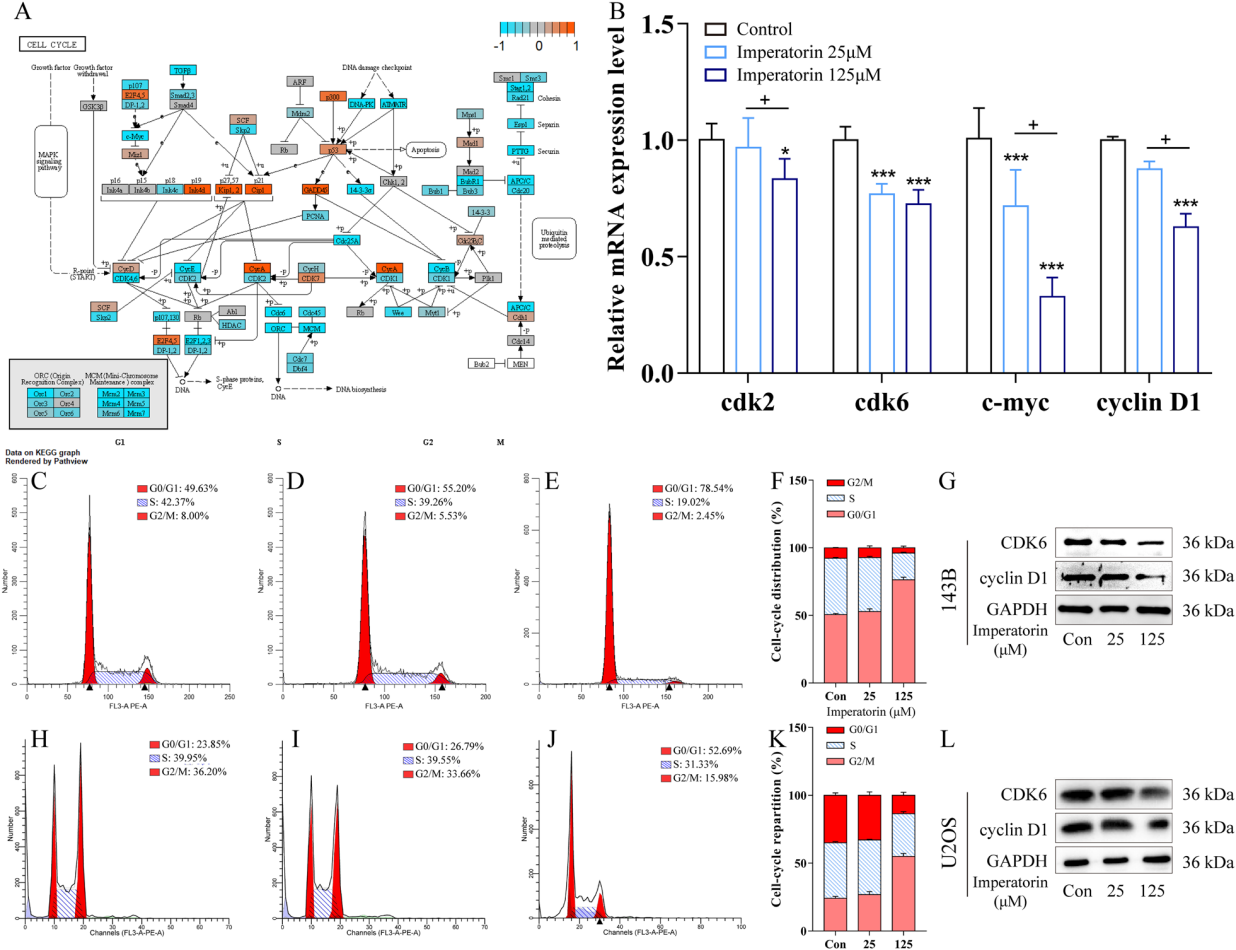
IMP induced G0/G1 phase arrest in cell cycle of human osteosarcoma cells. **A** A visualized cell cycle pathway heatmap with relative gene expression level. **B** The expression level of key genes in cell cycle pathway of 143B cells exposed to various concentration of IMP were determined by RT-PCR assay. The cell cycle distribution map of 143B cells treated with blank control (**C**), 25 μM IMP (**D**), 125 μM IMP (**E**) detected by cytometry, and the semi-quantitative statistical chart of them (**F**). The cell cycle distribution map of U2OS cells treated with blank control (**H**), 25 μM IMP (**I**), 125 μM IMP (**J**) detected by cytometry, and the semi-quantitative statistical chart of them (**K**). The expression level of key proteins CDK6 and Cyclin D1 of cell cycle pathway in 143B cells (**G**) and U2OS cells (**L**). *p  < 0.05; **p  < 0.01; ***p  < 0.001, one-way ANOVA; data were mean  ±  SD.^+^ p < 0.05;^++^ p < 0.01; ^+++^ p < 0.001, one-way ANOVA; data were mean ± SD

Cell cycle distribution analysis was conducted on OS cells exposed to various concentration of IMP to confirm its effects on cell cycle progress. As shown in Fig. 4C–F and Fig. 4H–K, IMP induced the accumulation of both 143B and U2OS cells in G0/G1 phase and a corresponding decrease of that in G2/M and S phase in a dose-dependent manner. In order to further elucidate the molecular mechanisms, a visualized cell cycle pathway heatmap with relative gene expression level was established according to the RNA-seq data (Fig. 4A), which demonstrated that a lot of key genes in cell cycle pathway, such as cdk2, cdk6, c-myc and cyclin D1, were down-regulated after IMP treatment. RT-PCR assay (Fig. 4B) of related genes was also performed to confirm the accuracy of RNA-seq results. Moreover, Western Blot assay results (Fig. 4G, L) showed that IMP treatment down-regulated the expression level of cell cycle key proteins Cyclin D1 and CDK6 in both 143B and U2OS cells. All these results confirmed that IMP induced the G0/G1 phase arrest by down-regulating the expression of key proteins of G0/G1 cell cycle regulator markers in OS cells.

### IMP triggered autophagy in vitro

**Fig. 5:**
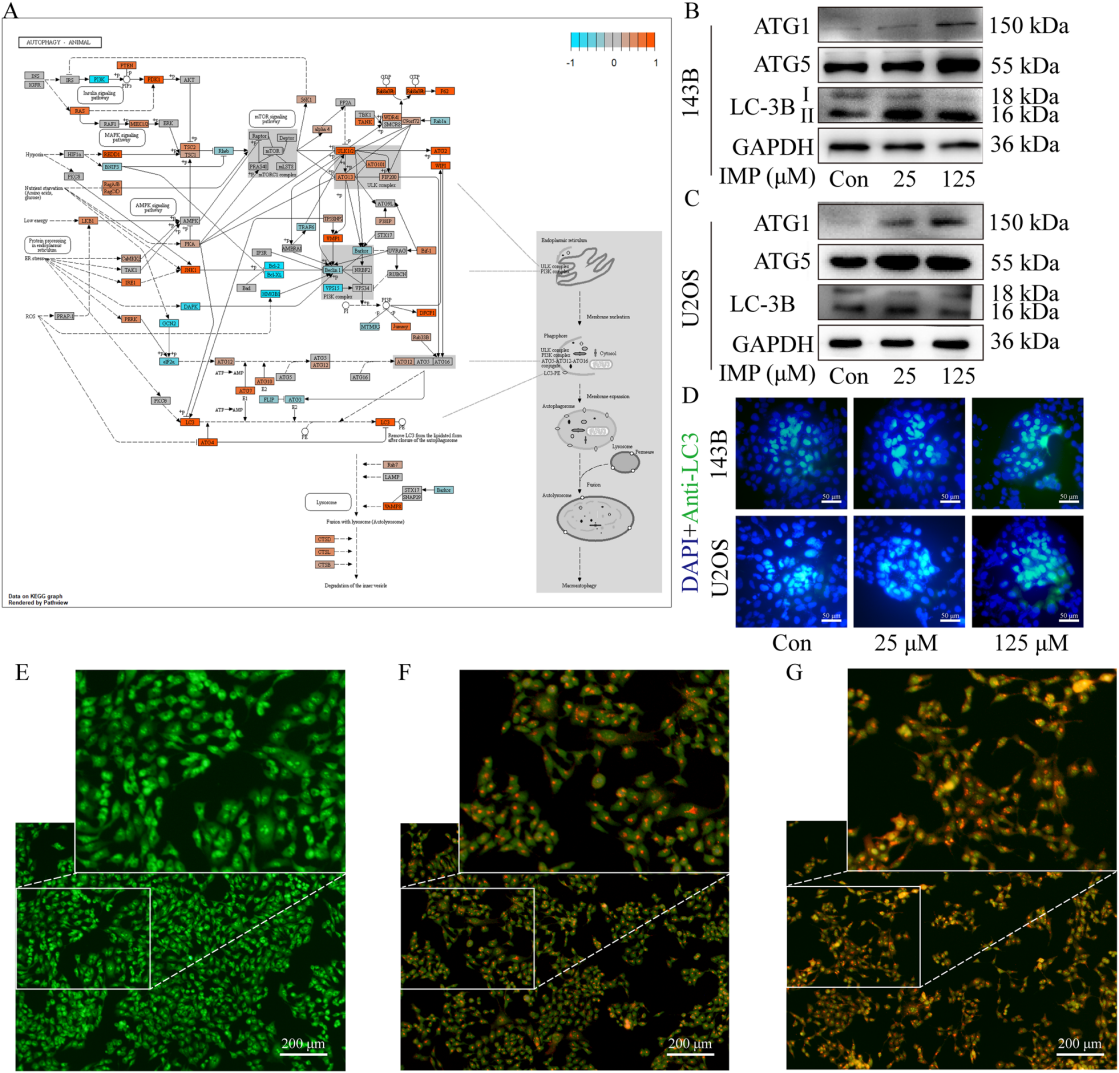
IMP triggered autophagy of human osteosarcoma cells. **A** A visualized autophagy pathway heatmap with relative gene expression level. The expression level of key proteins ULK1 (ATG1), ATG5 and LC3 in autophagy pathway of 143B cells (**B**) and U2OS cells (**C**) treated with various concentration of IMP were detected by Western Blot. **D** LC3B immunofluorescence images of 143B and U2OS cells treated with various concentration of IMP for 24 h. AO staining images of 143B cells treated with blank control (**E**), 25 μM IMP (**F**) and 125 μM IMP (**G**) were photographed by inverted fluorescence microscope. Scale bars in (**E**–**G**) equaled 200 μm while that in (**D**) equaled 50 μm

To understand the role of autophagy in IMP-induced cell death, acridine orange (AO) staining assay and LC3B immunofluorescence assay were conducted to determine the autophagy level of OS cells. As shown in Fig. 5D, IMP treatment significantly promoted the production of LC3B in both 143B and U2OS cells in a dose-dependent manner. Moreover, as shown in Fig. 5E–G, the accumulation of bright red acidic vesicles resembling autolysosomes was observed in IMP-treated 143B cells, in a dose-dependent manner. To further investigate the mechanisms of IMP-induced autophagy, a visualized autophagy pathway heatmap with relative gene expression level was also established according to the RNA-seq data (Fig. 5A). Moreover, the expression level of key proteins in autophagy process was measured by Western Blot assay. As shown in Fig. 5B, C; IMP treatment induced the significantly up-regulation of the expression level of ULK1(ATG1), ATG5 and the expression ratio of LC3-II to LC3-I, both in 143B and U2OS cells, which were consistent with the autophagy enhancement in IMP-treated OS cells. All these results confirmed that IMP treatment triggered cell autophagy by significantly up-regulating the expression level of some critical proteins in autophagy pathway.

### IMP suppressed PTEN-PI3K-AKT-mTOR/p21 signaling pathway by targeting and up-regulating PTEN in vitro

**Fig. 6:**
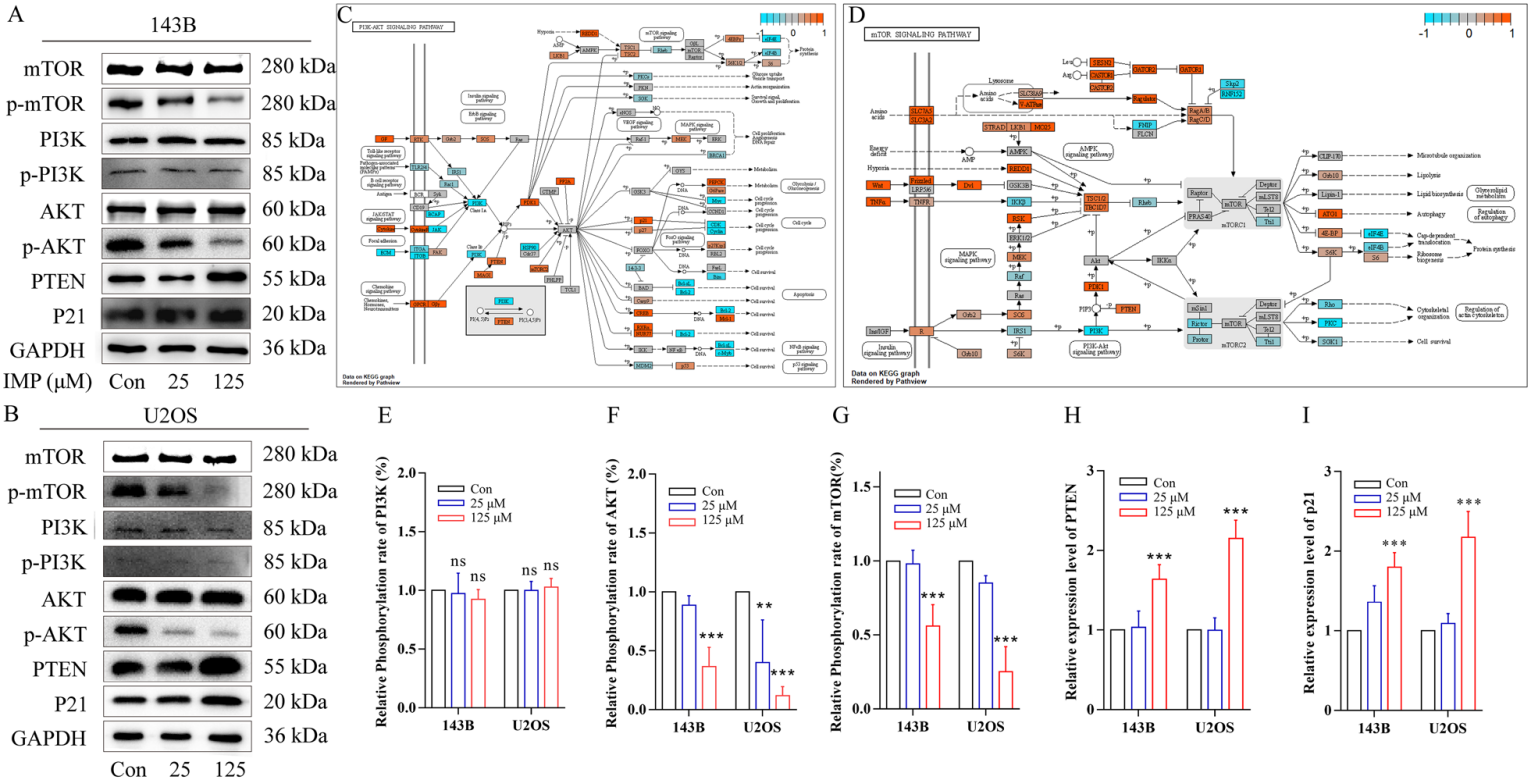
IMP suppressed PTEN-PI3K-AKT-mTOR/p21 signaling pathway. Visualized PI3K-AKT (**C**) and mTOR (**D**) KEGG pathway heatmap with relative gene expression level. The expression level of key proteins PTEN, PI3K, p-PI3K, AKT, p-AKT, mTOR, p-mTOR and p21 in PTEN-PI3K-AKT-mTOR/p21 pathway of 143B cells (**A**) and U2OS cells (**B**) exposed to various concentration of IMP and the semi-quantitative statistical charts of them(**E**–**I**) were determined by Western blot. ns p  ≥ 0.05; *p  < 0.05; **p  < 0.01; ***p  < 0.001, one-way ANOVA; data were mean  ±  SD

After we confirmed that IMP inhibited the cell proliferation mainly by the promotion of G0/G1 phase arrest and autophagy, visualized KEGG pathway heatmaps of PI3K-AKT and mTOR with relative gene expression level were established, based on the RNA-seq data, to further investigate the possible molecular mechanisms of IMP’s antitumor effect. As shown in Fig. 6C, D; IMP altered the expression level of many key genes in PI3K-AKT and mTOR signaling pathways. After considering the fold changes and biological function of a series of differential expressed genes, we assumed that IMP inhibited the activity of PTEN-PI3K-AKT-mTOR/p21 signaling pathway to induce the autophagy and G0/G1 phase arrest of OS cells. In order to confirm the role of PTEN-PI3K-AKT-mTOR/p21 pathway in IMP-induced autophagy and cell cycle arrest, western blot assay was performed to determine the expression level and phosphorylation level of the key regulatory proteins in PI3K-AKT-mTOR/p21 signaling pathway, including PI3K/p-PI3K, PTEN, AKT/p-AKT, p21 and mTOR/p-mTOR. As shown in Fig. 6A, B; IMP significantly up-regulated the expression level of PTEN, which subsequently inhibited PI3K-AKT-p21/mTOR signaling pathway that manifested itself in decreased phosphorylation level of AKT, mTOR, and increased expression level of p21. Besides, the phosphorylation of PI3K p85 subunit was not altered after IMP treatment. The results of western blot assay confirmed that, both in 143B and U2OS cells, IMP induced G0/G1 phase arrest and autophagy through the inhibition of PTEN-PI3K-AKT-mTOR/p21 pathway possibly via targeting and upregulating the expression of PTEN.

### Inhibition of PTEN rescued the effect of IMP towards OS cells in vitro

**Fig. 7:**
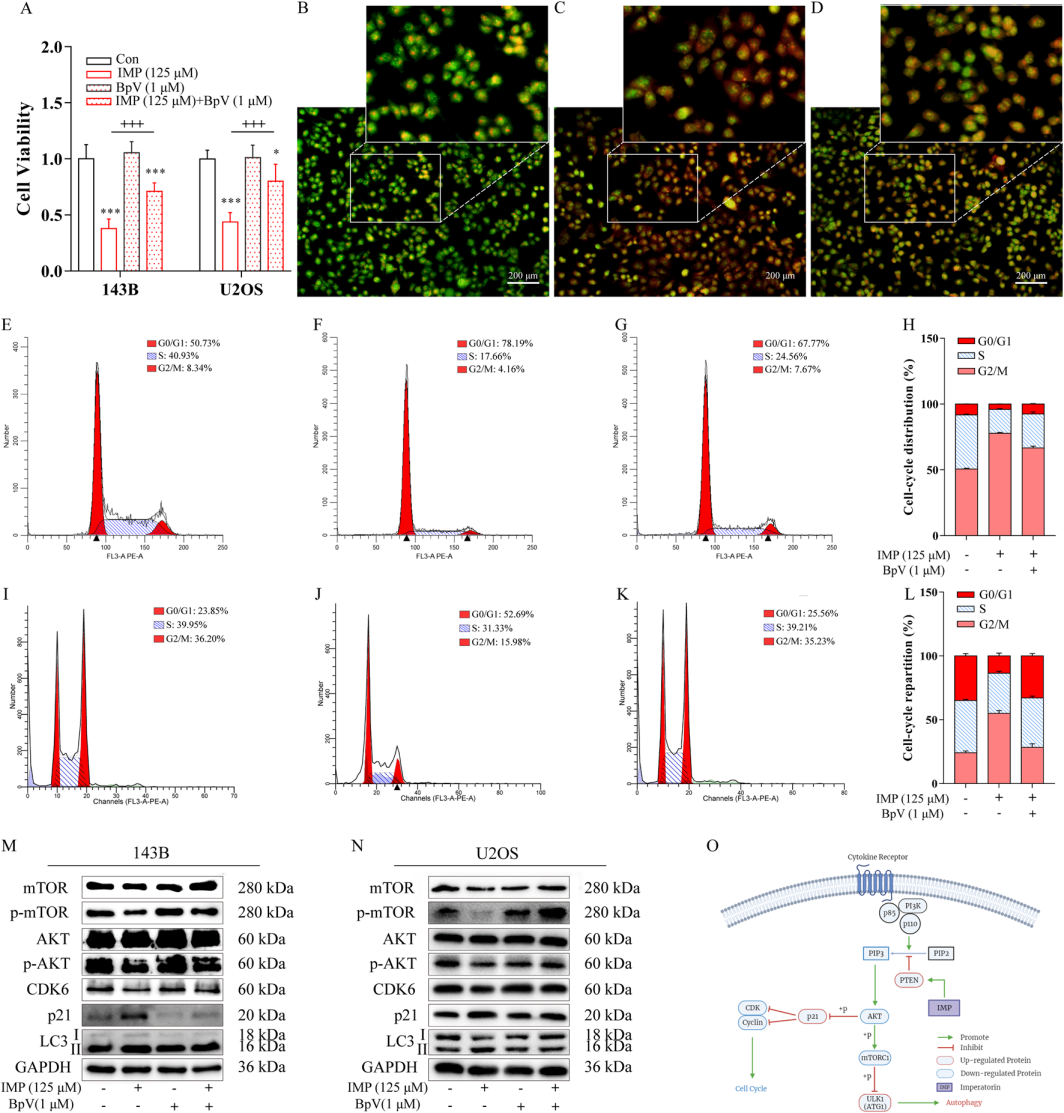
Inhibition of PTEN rescued the effect of IMP on human osteosarcoma cells. **A** Inhibition of PTEN reduced IMP-induced inhibitory effect on proliferation of 143B and U2OS cells. AO staining images of 143B cells treated with blank control (**B**), 125 μM IMP (**C**) and 125 μM IMP combined with 1 μM Bpv (HOpic) (**D**) were photographed by inverted fluorescence microscope. The cell cycle distribution map of 143B cells treated with blank control (**E**), 125 μM IMP (**F**), 125 μM IMP combined with 1 μM Bpv (HOpic) (**G**) detected by flow cytometry, and the semi-quantitative statistical chart of them (**H**). The cell cycle distribution map of U2OS cells treated with blank control (**I**), 125 μM IMP (**J**), 125 μM IMP combined with 1 μM Bpv (HOpic) (**K**) detected by flow cytometry, and the semi-quantitative statistical chart of them (**L**). The expression level of key proteins AKT, p-AKT, mTOR, p-mTOR, p21, CDK6 and LC3 in 143B cells (**M**) and U2OS cells (**N**) exposed to various concentration of IMP and Bpv were determined by Western blot. **O** A schematic of the effects of IMP on PTEN-PI3K-AKT-mTOR/p21 signaling pathway in human osteosarcoma cells. Scale bars equaled 200 μm. *p  < 0.05; **p  < 0.01; ***p  < 0.001; one-way ANOVA; ^ +^  p  < 0.05;  ^++^  p  < 0.01;  ^+++ ^ p  < 0.001, one-way ANOVA; data were mean  ±  SD

To further confirm that IMP-induced up-regulation of PTEN was the primary cause of the following suppression of PI3K-AKT-mTOR/p21 signaling pathway, Bpv (HOpic), a potent and selective inhibitor of PTEN, was applied to conduct a rescue experiment (Fig. 7). The cell viability assay demonstrated that, as shown in Fig. 7A, the presence of Bpv (1 μM) significantly rescued IMP-induced cell death (p  < 0.05), both in 143B and U2OS cells. AO staining assay (Fig. 7B–D) illustrated that the number of bright red acidic vesicles was apparently reduced after the combination of IMP and Bpv treatment, compared with IMP-treated group. As for cell cycle distribution (Fig. 7E–L), Bpv partially counteracted the IMP-induced accumulation of OS cells in G0/G1 phase. In terms of protein expression (Fig. 7M, N), the combination of IMP and Bpv treatment significantly increased expression level of CDK6, elevated the phosphorylation level of AKT, mTOR and decreased the expression level of p21, compared with the IMP group, which confirmed that Bpv apparently rescued the IMP-induced inhibition of PI3K-AKT-mTOR/p21 signaling pathway in both 143B and U2OS cells. All these results confirmed that inhibition of PTEN significantly rescued the effects of IMP towards OS cells, including the influence on cell autophagy, cell cycle distribution, and the activity of PI3K-AKT-mTOR/p21 signaling pathway, which suggested that IMP triggered antitumor effect through targeting and up-regulating the expression level of PTEN. Figure 7O illustrated the mechanisms of IMP’s effect on human osteosarcoma cells.

### IMP inhibited osteosarcoma growth in vivo

**Fig. 8:**
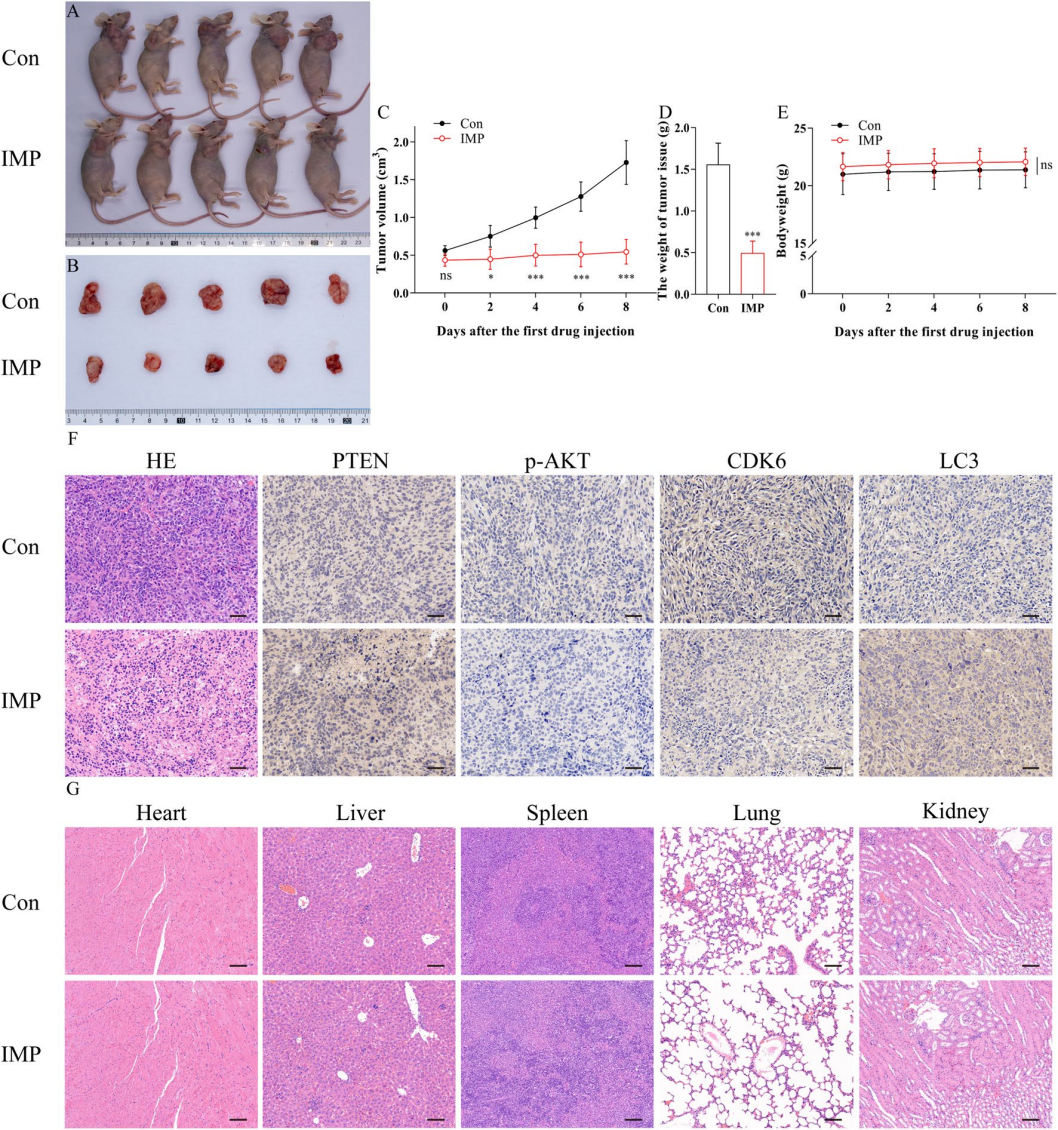
IMP inhibited osteosarcoma growth in vivo. **A**–**C** IMP treatment significantly reduced the tumor volume in subcutaneous tumor-transplanted nude mouse model. **D** IMP treatment decreased the weight of tumor issue in tumor-transplanted nude mouse model. **E** IMP treatment did not alter the bodyweight of animals, compared with the control group. **F** H&E staining and IHC staining against PTEN, p-AKT, CDK6 and LC3 images on tumor tissues after the treatment of each group. **G** H&E staining images of vital organs including heart, liver, spleen, lung and kidney of animals in each group. Scale bars equaled 50 μm in **E** and equaled 100 μm in **F**. ns p  ≥  0.05; *p  < 0.05; **p  < 0.01; ***p  < 0.001, one-way ANOVA; data were mean  ±  SD

To evaluate the internal effect of IMP on osteosarcoma, a subcutaneous tumor-transplanted nude mouse model was established. IMP at dose of 5 mg·kg^−1^ was intraperitoneal injected every other day. As shown in Fig. 8A–D, after five times of injection, 5 mg·kg^−1^ IMP treatment significantly decreased the tumor weight and volume, compared with the control group (p  < 0.05). However, there was no obvious bodyweight difference observed between the IMP and the control group (Fig. 8E), which suggested the low systematic toxicity of IMP treatment. Besides, the immune-histochemistry staining images (Fig. 8F) illustrated that the IMP-treated tumor tissues exhibited significantly increase in PTEN- and LC3-positive cells, as well as p-AKT- and CDK6-negative cells, which were consistent with the results of external experiments. To further investigate the potential toxicity of IMP on normal tissues in vivo, the vital organs including heart, liver, spleen, lung and kidney of the nude mouse models were also collected at the end of the animal experiment. The H&E staining of these organs (Fig. 8G) revealed no obvious toxicities of IMP on them. These data suggested that IMP at dose of 5 mg·kg^−1^ exhibited desirable antitumor activity as well as low toxicity in vivo. Moreover, the IHC results confirmed our conclusion of molecular mechanisms of IMP’s effects on human osteosarcoma cells in vivo.

## Discussion

In recent years, owing to the rapid development of new therapeutic methods, the 5-year survival rate of early or localized osteosarcoma has been promoted to above 70% [4]. Whereas, the long-term prognosis of osteosarcoma, especially for unresectable and metastatic tumors, still remains unsatisfactory in the past decades [5]. Hence, it’s very necessary to develop new therapeutic agents which exhibit effective and selective antitumor effects. In this work, we systematically evaluated the anti-tumor effect of imperatorin on osteosarcoma cells, demonstrated that autophagy and G0/G1 phase arrest were the two main causes of IMP-induced cell death. Moreover, we assumed and confirmed that IMP inhibited the activity of PTEN-PI3K-AKT-mTOR/p21 signaling pathway to trigger autophagy and cell cycle arrest of OS cells by up-regulating the expression level of PTEN in vitro and in vivo (as shown in Fig. 7O).

The transition from G0/G1 phase to S phase of cell cycle is crucial for the control of cell proliferation, while its misregulation usually leads to oncogenesis [25]. It has been reported that G1-S transcriptional activation at late G1 phase resulted in the commitment of cells to enter the cell cycle and cell proliferation [33, 34]. At early G1 phase, pocket proteins RB, p107 and p130 are bound to E2F (E2F1, E2F2, E2F3) transcription factors, the activators of G1-S transcription, to blocks E2F’s transcription and expression [35–39]. The phosphorylation of pocket proteins by cyclinD1-CDK4 and cyclinD1-CDK6 releases them from E2F transcription factors, which allows the initial activation of G1-S transcription, including genes encoding cyclin E [40]. Cyclin E-CDK2 can also phosphorylate the pocket proteins, thereby forming a positive feedback loop which ensures the cell cycle commitment [25]. In our work, it has been proved that IMP treatment inhibited G1-S transition and down-regulated the expression level of genes including cdk2, cdk4, cdk6 and cyclin D1, cyclin E, as well as that of proteins including Cyclin D1 and CDK6. These results suggested that IMP treatment inhibited G1-S transcription and thereby suppressed G1-S transition by breaking the positive feedback loop after the pocket protein phosphorylation via inactivating cyclin-CDK complex.

Autophagy is a self-degradative progress of cells which contains three defined types: macro-autophagy, micro-autophagy, and chaperone-mediated autophagy [41]. On molecular level, this complex process has three main regulable steps: phagophore formation, ATG5-ATG12 conjugation, and LC3 processing and insertion into the phagophore membrane. It has been reported that the activity of ATG1 (ULK1)-ATG13-ATG17 complex, which is regulated by mTOR that can inhibits the autophagy by phosphorylating ATG13, is possibly essential for initiating phagophore formation [24, 42–44]. Moreover, in ATG5-ATG12 conjugation step, ATG12 and ATG5 are covalently conjugated with the help of ATG7 and ATG10, which is part of the vesicle elongation process [24]. In LC3 processing step, LC3-I is activated and transferred to ATG3 to generate processed LC3-II in the phagophore, which is dependent on ATG5-ATG12 complex [45]. Therefore, the expression ratio of LC3-II to LC3-I is identified as a key readout of autophagy level in cells. In our work, the result of AO staining, LC3 immunofluorescence, and western blot assay together demonstrated that IMP treatment resulted in an increased level of autophagy in OS cells, as well as an increased expression level of key proteins including ULK1 (ATG1), ATG5 in autophagy process. These data suggested that IMP possibly altered the activity of mTOR and thereby activated ULK1 (ATG1)–ATG13–ATG17 complex to initiate the autophagy process. However, the mechanism of IMP-induced up-regulation of ATG5 was still unclear and needed further investigation.

PI3K-AKT signaling pathway contributed to many biological processes, especially for the growth and survival of various kinds of tumors. PI3K (class IA) consists of a p85 regulatory subunit and a p110 catalytic subunit [27]. p85 subunit can be phosphorylated by upstream receptor tyrosine kinases to thereby recruit p110 subunit to the membrane, which can generate 3′ phosphoinositide lipids (PIP3) that usually function as second messengers [46]. This phosphorylating process is directly regulated by PTEN, a vital regulator that is able to dephosphorylate PIP3 to antagonize PIP3 generation [47]. Moreover, it has been reported that the generation of PIP3 transfers the signals from extracellular cytokines, growth factors, and environmental stimuli into intracellular messages which thereby activate the downstream effectors, such as AKT [48]. AKT is a main PI3K effector that is capable of activating mTOR (C1) signaling pathway [49]. Then, mTORC1 negatively regulated a critical Ser/Thr kinase ULK1 (ATG1) [50], which can initiate the autophagy process as mentioned above. Moreover, the activation of PI3K-AKT pathway has also been reported that it downregulates the expression of p21, a member of CIP/KIP family that is able to down-regulate the activity of cyclin-CDK complex to alter the cell cycle distribution as mentioned above [51–53]. In our work, the western blot data suggested that IMP up-regulated the expression of PTEN and p21, concurrently down-regulated the phosphorylation of AKT and mTOR. What’s more, the inhibition of PTEN by 1 μM Bpv (HOpic) treatment significantly rescued a series of antitumor effect of IMP on OS cells. Therefore, all these results demonstrated that IMP targeted and up-regulated the expression of PTEN, which thereby inhibited PIP3 generation and AKT phosphorylation, sequentially. Then, the repression of PI3K-AKT pathway inactivated mTOR as well as concurrently activated p21, which lead to autophagy promotion and G0/G1 phase arrest in cell cycle, respectively.

Furthermore, it has been reported that the usage of rapamycin, an antitumor agent targeting the inactivation of mTOR, usually leads to drug resistant against that in many kinds of tumors [54]. This phenomenon occurred because the long-term inhibition of mTOR repressed the phosphorylation of downstream protein p70S6K1, which thereby induced the activation of AKT (the upstream regulator of mTOR) via the relief of p70S6K1-RTK-AKT negative feedback loop [54]. According to our results, IMP was able to inhibit the activation of AKT through PTEN-AKT pathway. We believed that the combination usage of IMP and rapamycin was likely to reverse the drug resistance against rapamycin and synergically enhance their antitumor effect, which needs further investigation in the future.

## Conclusion

In this study, the results demonstrated that imperatorin was a promising anti-tumor agent with a series of repressive effects on osteosarcoma cells, including cell proliferation, invasion and migration. Imperatorin significantly inhibited the growth of osteosarcoma through G0/G1 phase arrest and autophagy promotion in vitro and in vivo. Moreover, the results confirmed imperatorin induced cell cycle and autophagy alteration by inhibiting the activity of PTEN-PI3K-AKT-mTOR/p21 signaling pathway through targeting and up-regulating the expression of PTEN. All these findings suggested that imperatorin was a desirable candidate for clinical treatments on osteosarcoma.

## Supplementary Information

**Additional file 1: Table S1.** Primer sequences for real-time PCR

## Data Availability

The raw data of RNA-seq assay in this work has been submitted to public database GEO (No. GSE180321).
