# Supplementary material for: Imperatorin induces autophagy and G0/G1 phase arrest via PTEN-PI3K-AKT-mTOR/p21 signaling pathway in human osteosarcoma cells in vitro and in vivo
Source: Cancer Cell Int. 2021 Dec 19;21:689. doi: 10.1186/s12935-021-02397-7 (PMC8684670; doi:10.1186/s12935-021-02397-7)
Supplement: Supplementary file 1 — Additional file 1: Table S1. Primer sequences for real-time PCR [file 12935_2021_2397_MOESM1_ESM.docx]

**Imperatorin induces autophagy and G0/G1 phase arrest via PTEN-PI3K-AKT-mTOR/p21 signaling pathway in human osteosarcoma cells in vitro and in vivo**

Minchao Lv^1^, Bei Zhang^2^, Zhiqiang Yang^3^, Jun Xie^1^, Jinku Guo^1^, Feixiong He^1^#, Wei Wang^1^#.

# Supplementary Information

Number of Tables: 1

Number of Figures:0

**Table S1**. Primer sequences for real-time PCR

| Gene | Forward Sequence | Reverse Sequence |
| --- | --- | --- |
| β-actin | 5’‐CTGGAGCATGCCCGTATTTA‐3’ | 5’‐TTTGGTCTTGCCACTTTTCC‐3’ |
| CDK2 | 5’- TTTGCTGAGATGGTGACTCG -3’ | 5’-CTTCATCCAGGGGAGGTACA-3’ |
| CDK6 | 5’-GGCTGTGTGAACCAGCCCAAG-3’ | 5’-TGGCCAGGCCTAGACAGGCA-3’ |
| c-myc | 5’-TCAAGAGGTGCCACGTCTCC-3’ | 5’-TCTTGGCAGCAGGATAGTCCTT-3’ |
| Cyclin D1 | 5’-AGAAGGAGGTCCTGCCGTCC-3’ | 5’-GGTCCAGGTAGTTCATGGCC-3’ |
